# Microglia and Neuroinflammation: Crucial Pathological Mechanisms in Traumatic Brain Injury-Induced Neurodegeneration

**DOI:** 10.3389/fnagi.2022.825086

**Published:** 2022-03-25

**Authors:** Fangjie Shao, Xiaoyu Wang, Haijian Wu, Qun Wu, Jianmin Zhang

**Affiliations:** ^1^Department of Plastic and Aesthetic Center, The First Affiliated Hospital, School of Medicine, Zhejiang University, Hangzhou, China; ^2^Department of Neurosurgery, The Second Affiliated Hospital, School of Medicine, Zhejiang University, Hangzhou, China; ^3^Brain Research Institute, Zhejiang University, Hangzhou, China; ^4^Collaborative Innovation Center for Brain Science, Zhejiang University, Hangzhou, China

**Keywords:** microglia, neuroinflammation, traumatic brain injury, neurodegeneration, central nervous system

## Abstract

Traumatic brain injury (TBI) is one of the most common diseases in the central nervous system (CNS) with high mortality and morbidity. Patients with TBI usually suffer many sequelae in the life time post injury, including neurodegenerative disorders such as Alzheimer’s disease (AD) and Parkinson’s disease (PD). However, the pathological mechanisms connecting these two processes have not yet been fully elucidated. It is important to further investigate the pathophysiological mechanisms underlying TBI and TBI-induced neurodegeneration, which will promote the development of precise treatment target for these notorious neurodegenerative consequences after TBI. A growing body of evidence shows that neuroinflammation is a pivotal pathological process underlying chronic neurodegeneration following TBI. Microglia, as the immune cells in the CNS, play crucial roles in neuroinflammation and many other CNS diseases. Of interest, microglial activation and functional alteration has been proposed as key mediators in the evolution of chronic neurodegenerative pathology following TBI. Here, we review the updated studies involving phenotypical and functional alterations of microglia in neurodegeneration after injury, survey key molecules regulating the activities and functional responses of microglia in TBI pathology, and explore their potential implications to chronic neurodegeneration after injury. The work will give us a comprehensive understanding of mechanisms driving TBI-related neurodegeneration and offer novel ideas of developing corresponding prevention and treatment strategies for this disease.

## Introduction

Traumatic brain injury (TBI) represents one of the most common causes of death and disability that can affect people of all ages (Maas et al., 2017). It is estimated that 69 million individuals will suffer TBI each year worldwide. Based on clinical factors and severity, it can be categorized into mild (∼80%), moderate (∼10%), and severe (∼10%) cases (Dewan et al., 2018). In addition to the acute risk of morbidity with moderate to severe injuries, traumatic brain injury is associated with a number of chronic neurological and neuropsychiatric sequelae including neurodegenerative diseases such as Alzheimer’s disease (AD), Parkinson’s disease (PD), and chronic traumatic encephalopathy (CTE) (Wilson, 2016; Wilson et al., 2017). However, despite the high incidence of TBI and its chronic and evolving neurodegenerative consequences, the potential causative factors linking these two processes have not yet been fully elucidated. It is imperative to further explore the pathophysiological mechanisms underlying TBI and its related neurodegeneration, which enables the development of potential cellular and molecular targets for these notorious neurodegenerative processes.

Pathophysiologically, damage to brain tissue after TBI can be classified as primary and secondary brain injury. The primary injury occurs as a direct result of the initial physical forces; while the secondary injury arises from pathophysiological cascades following the traumatic episode (Blennow et al., 2016). Such secondary processes typically evolve over time and can occur up to days, weeks or even years after the initial traumatic event, which provides a time window for therapeutic intervention (Cruz-Haces et al., 2017; Wilson et al., 2017). Of note, these damages are frequently mediated by multiple mechanisms such as glutamatergic excitotoxicity, free radicals and reactive oxygen species (ROS) overproduction, neuroinflammation, apoptosis and blood–brain barrier (BBB) dysfunction. Among them, neuroinflammation is conceived as one of the most common but not well-characterized pathophysiological events in the pathogenesis of TBI (Jassam et al., 2017). It has the ability to mediate secondary injury and neurodegeneration versus promote neural repair and regeneration after TBI (Simon et al., 2017). In particular, increasing evidence suggests neuroinflammation is a potentially key orchestrator of chronic neurodegenerative processes after TBI (Jassam et al., 2017; Simon et al., 2017). The causative roles of neuroinflammatory responses in TBI-related neurodegeneration are under investigation and demand further elucidation.

Microglia, the resident immune cells of the central nervous system (CNS), account for ∼10% of the total cell population in the adult brain, with variable cell density across distinct anatomical regions (Lawson et al., 1990, 1992; Mittelbronn et al., 2001). They are the most abundant immune sentinels within the CNS and play critical roles in brain health and diseases (Wolf et al., 2017). Under physiological conditions, microglia exhibit a myriad of activities that allow them to sense and screen surrounding inflammatory cues, promote synaptic remodeling and neuronal function, as well as phagocytose and remove cellular debris (Prinz et al., 2019; Cserép et al., 2020). Under pathological conditions, microglia can be activated and play crucial roles in neuroinflammation and CNS diseases, ranging from acute injuries such as stroke and TBI to chronic neurodegeneration such as AD, PD, and CTE (Karve et al., 2016; Kim and Cho, 2016; Colonna and Butovsky, 2017). Of interest, microglial activation and functional alteration has been proposed as key mediators in the evolution of chronic neurodegenerative pathology following TBI (Morganti et al., 2016; Jassam et al., 2017). Elucidating the long-term changes of microglial physiology after TBI will facilitate our understanding of pathological links between TBI and chronic neurodegenerative disease.

Here, we review the evidence of TBI-induced alterations in microglial phenotypes and functions, focusing specifically on their involvement in neurodegeneration after injury. Deciphering their unique contributions to TBI-related neurodegeneration in a disease-stage dependent manner is particularly significant, which enables us to uncover the complexity of targeting microglia for developing precise prevention and treatment strategies for these notorious neurodegenerative consequences following TBI.

## Heterogeneity of Microglia: Phenotypic and Functional Diversity

**FIGURE 1:**
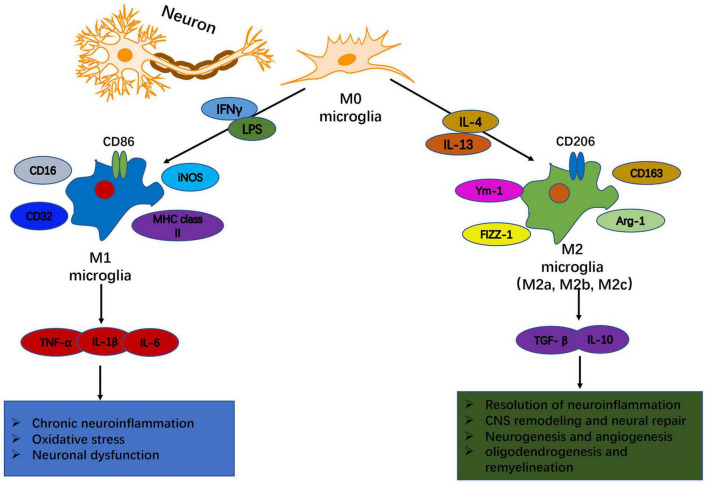
Heterogeneity of microglia: phenotypic and functional diversity. In response to disturbance of brain homeostasis like injury or disease, microglia can become polarized toward M1-like and M2-like activation states that can have distinct roles in neurodegeneration and tissue repair. An environment rich with the classical pro-inflammatory stimuli, such as IFN-γ and LPS, promotes the polarization of resting microglia into an M1 phenotype. M1-like microglia are characterized by upregulated expression of phenotypic protein markers such as IL-1β, TNFα, IL-6, and iNOS. They release pro-inflammatory cytokines, chemokines and free radicals that impair brain repair and contribute to chronic neuroinflammation, oxidative stress and long-term neurological impairments. In contrast, a neuroinflammatory environment rich in anti-inflammatory IL-4 or IL-13 drives the development of an M2 phenotype. M2-like microglia upregulate protein markers such as CD206, CD163, FCγR, arginase 1, Ym-1, and TGFβ. M2-like microglia release anti- inflammatory cytokines, neurotrophic factors and proteases, and they have increased phagocytic activity. M2-like microglia promote immunosuppression and resolution of M1-mediated neuroinflammation, and participate in CNS remodeling and repair by modulating neurorestorative processes such as neurogenesis, angiogenesis, oligodendrogenesis and remyelination.

Microglia are most abundant CNS innate immune sentinels residing in the parenchyma. They are endowed with spectacular plasticity, which enables them to acquire multiple phenotypes and thereby fulfill numerous functions in health and disease (Figure 1; Stratoulias et al., 2019). In the healthy mature CNS, microglia retain a highly ramified morphology of a small soma with fine cellular processes (Kettenmann et al., 2011). These “resting” cells actively survey surrounding microenvironment without disturbing the neurovascular coupling. Upon disturbance of brain homeostasis like injury or disease, microglia can evoke rapid and profound changes in the cell shapes, gene expressions and functional behaviors, which are defined as “microglial activation” (Kettenmann et al., 2011). Besides, other factors including aging, lifestyle, diet, sleep pattern, stress, and environmental factors, have been identified closely associated with microglial activity and degree of microglia-induced neuroinflammation (Madore et al., 2020; Popa-Wagner et al., 2020). Activated microglia can migrate toward a lesion and phagocytose of cellular debris or damaged cells, orchestrate neuroinflammation by secreting important inflammatory mediators, as well as release numbers of substances (i.e., neurotrophic factors and reactive oxygen species) that can exert beneficial or detrimental effects on surrounding cells (Wolf et al., 2017). It is now appreciated that microglia are not passive bystanders that merely react to brain pathology, but instead, exert more active roles in the pathophysiology of numerous CNS diseases (Wright-Jin and Gutmann, 2019).

Traditionally, activated microglia can be categorized into distinct phenotypes based on their cellular morphology, surface marker expression, and physiological properties (Prinz et al., 2019). As an example, the M1-M2 dichotomy has been proposed for microglial activation in experimental systems: classical M1 vs. alternative M2, respectively (Franco and Fernández-Suárez, 2015). The classical M1 microglia exhibit pro-inflammatory and neurotoxic activities and express high levels of cellular markers such as CD16, CD32, CD86, iNOS and MHC class II (Orihuela et al., 2016). By contrast, the alternative M2 microglia, which are characterized by the expression of phenotypic markers such as CD163, CD206, Arg-1, Ym-1, and FIZZ-1, play a role in inflammatory dampening, debris scavenging and tissue remodeling (Mecha et al., 2016). Based on their distinct molecular signatures and functional profiles, M2 microglia can be further subclassified into M2a, M2b, M2c, among others (Cherry et al., 2014). The M2a-like phenotype secretes anti-inflammatory cytokines and growth factors as well as increases phagocytic activity, which contributes to inflammation resolution and tissue repair; the M2b-like phenotype, a type II alternative activation, is associated with immunosuppression and tissue homeostasis; and the M2c-like phenotype, an acquired deactivation, is involved in immune regulation, tissue repair and remodeling (Simon et al., 2017). Of note, the M1/M2 category is helpful to conceptualize microglia activities in *in vitro* models, however, it is an oversimplified conceptual framework and is inadequate to describe microglial activation in *in vivo* conditions (Hu et al., 2015). Indeed, the status of microglia *in vivo* is far more complicated than *in vitro*, which may include a spectrum of different but functionally overlapping phenotypes.

More recently, the advent of single-cell technologies has provided new insights into the much more complex and fascinating biology of microglia in normal and disease processes (Sankowski et al., 2019; Masuda et al., 2020). In particular, the single-cell RNA-sequencing (RNA-Seq) has untangled the diversity of microglia during development, homeostasis, and disease in both mice and humans (Hammond et al., 2019; Li et al., 2019; Tan et al., 2020). Emerging evidence provides support to the existence of putative diverse microglial subpopulations endowed with unique genomic, morphological, spatial and functional specializations, such as homeostatic microglia (Sousa et al., 2018), proliferative region-associated microglia (Li et al., 2019), axon tract-associated microglia (ATM) (Hammond et al., 2019), disease-associated microglia (Keren-Shaul et al., 2017), recovery-associated microglia (Tay et al., 2018), among others. And a combination of massively parallel single-cell analysis, highly multiplexed single-molecule fluorescence *in situ* hybridization, advanced immunohistochemistry and computational modeling would empower the comprehensively characterization of diverse subclasses of microglia in multiple regions of the CNS during homeostasis and disease (Shah et al., 2016; Masuda et al., 2019).

## Microglial Activation and Its Dual Role in Traumatic Brain Injury

As the immune-competent cells of the brain, microglia play an increasingly crucial role in maintaining a healthy brain and have significant pathological implications in neurological diseases (Wolf et al., 2017). After TBI, these never-resting cells usually respond within minutes toward the sites of damage and manipulate neuroinflammatory responses and secondary cascades after injury (Kumar et al., 2017; Wofford et al., 2017; Lee S. W. et al., 2019). Moreover, sustained microglial alterations are involved in chronic neuroinflammation and long-term neuropathology after TBI (Wilson et al., 2004; Ritzel et al., 2020). The microglial responses to TBI include multiple distinct characteristics that no less than morphological transformation, electrophysiological changes, proliferation, migration, phagocytosis and secretory activities (Jassam et al., 2017). And they are not inherently neurotoxic but can also be neuroprotective following TBI (Loane and Kumar, 2016). Actually, their contributions to TBI lesions should be considered temporally and contextually, which should be taken into consideration during the development of therapeutics to regulate microglia-related inflammatory processes at specific stages of TBI.

## Microglial Responses in Human Traumatic Brain Injury

Microglial activation is recognized to be a key cellular mediator in the pathophysiology of TBI (Loane and Kumar, 2016). Evidence from clinical studies demonstrated that activated microglia are frequently expressed in the brain at the acute phase (Beschorner et al., 2002; Schwab et al., 2002; Bohnert et al., 2020), the sub-acute phase (Oehmichen et al., 1999; Engel et al., 2000), and even the chronic phase after the initial brain trauma, particularly following moderate to severe TBI (Ramlackhansingh et al., 2011; Johnson et al., 2013). For instance, immunohistochemical assays revealed that TMEM119^+^ microglial cells are abundantly expressed in the region of the cerebral cortex and the surrounding white matter after TBI, even in those cases with survival time less than 2 h (Bohnert et al., 2020). Also, Schwab et al. (2002) found that cyclooxygenase-1 (COX-1)-expressing microglia/macrophages are already evident in the injured brain within 6 h post-injury, reaching maximal levels after several weeks and remaining elevated at submaximal levels for several months after injury. Beschorner et al. (2002) reported that CD14^+^ microglia/macrophages, both at the lesion and in adjacent perilesional areas, are significantly increased within 1–2 days and can persist for months following closed TBI. Specifically, with survival of ≥3 months from injury, a proportion of cases with TBI displayed extensive, densely packed, CR3/43^+^ and/or CD68^+^ reactive microglia, a pathology not seen in control subjects or acutely injured cases (Johnson et al., 2013). This inflammatory pathology could still be observed up to 18 years after just a single TBI, along with ongoing white matter degradation post-injury (Johnson et al., 2013). Notably, in addition to immunohistochemical methods, neuroimaging techniques such as positron emission tomography (PET) and single photon emission computed tomography (SPECT) have also facilitated the investigation of microglial activation in human beings following TBI. As an example, microglial activation inferred by the *in vivo* PET imaging of [11C](R)-PK11195 binding is significantly raised in multiple subcortical regions in moderate to severe TBI survivors, including the thalami, putamen, occipital cortices, and posterior limb of the internal capsules (Ramlackhansingh et al., 2011). It suggests that these enhanced inflammatory responses can be detected up to 17 years after TBI (Ramlackhansingh et al., 2011). Besides, data from a longitudinal ^123^I-CLINDE SPECT study indicated that microglial activation and neuroinflammation are dispersed throughout the brain in mild TBI (mTBI) patients without signs of structural damage both at 1–2 weeks and 3–4 months after injury, even in those patients with good recovery (Ebert et al., 2019). All together, these evidences imply that microglia are persistently activated after TBI, emphasizing that this disease should not be viewed as a static insult, but instead as a trigger for a complex cascade of events evolving over time, including ongoing neuroinflammatory responses.

## Microglial Activities in Experimental Traumatic Brain Injury

In view of the heterogeneous nature of the clinical situations in TBI, numerous animal models have been developed to mimic this disease over the past decades (Xiong et al., 2013). Among them, cortical impact injury (CCI), fluid percussion injury (FPI), weight drop injury and blast injury are widely used in TBI research (Ma et al., 2019). These models have aided us tremendously to understand the pathological events after TBI, including microglial activation and neuroinflammation (Jassam et al., 2017). And evidence from preclinical studies indicated that rapid microglial reactivity is already detected within minutes in animal models of both mild and severe TBI (Davalos et al., 2005; Wofford et al., 2017). This process can last for days, weeks, and even months after experimental TBI, depending on different types of lesions (Cao et al., 2012; Robinson et al., 2017; Ritzel et al., 2020). More interestingly, phenotypic analysis of the temporal dynamics of microglia/macrophage polarization following TBI demonstrated that both M1-like and M2-like polarized microglia/macrophages are activated during the early stage after injury (Wang et al., 2013; Kumar et al., 2016a). However, the M2-like responses become dissipated over time, resulting in development of pathological M1-like phenotypes, which may eventually elicit secondary expansion of injury after trauma (Wang et al., 2013; Kumar et al., 2016a). Thus, inhibiting M1 phenotype while inducing M2 phenotype activation of microglia/macrophages by regulating key molecules such as NADPH oxidase 2 (NOX2) (Wang et al., 2017), histone deacetylases (HDACs) (Wang et al., 2015), high-mobility group box 1 (HMGB1) (Gao et al., 2018) and transforming growth factor-β1 (TGF-β1) (Zhao et al., 2020) can modify inflammatory responses, reduce neuronal damage, alleviate white matter injury and facilitate neurological functional recovery after TBI. Multiple types of drugs targeting microglia have been applied in experimental models of TBI to alleviate neuronal damage. For instance, SB 290157, as an antagonist of complement C3a receptor (C3aR) expressed in microglia, was intracortically administered in a mouse model. The researchers found that the number of inflammatory microglial cells migrating toward the lesion was diminished, and the capability of microglial phagocytosis was decreased (Surugiu et al., 2019).

Undoubtedly, the M1/M2 classification for microglia phenotyping based on the expression of a limited number of molecular markers is unlikely to reflect the true diversity of microglial function in TBI. In this regard, novel techniques such as single cell RNA-Seq provide unprecedented opportunities for establishing definitive profiles of microglia during different stages of TBI. By using the unbiased single cell sequencing method Drop-seq, Arneson et al. (2018) identified >50 differentially expressed genes (DEGs) within the microglial cluster in hippocampus between sham and mTBI animals at 24 h post-trauma, which are proposed to be linked with inflammation/immune responses after TBI. Furthermore, transcriptome analysis of microglia isolated from injured brain by fluorescence-activated cell sorting (FACS) up to 2 months after CCI demonstrated time-dependent changes in microglia transcriptional networks following TBI (Izzy et al., 2019). Specifically, there is a time-dependent, injury-associated dysregulation in the microglial ability to sense tissue damage, perform housekeeping, and maintain homeostasis in the early stages after CCI (Izzy et al., 2019). These immune responses resolve over time with transition to a pro-inflammatory state in the chronic stage of the injury (Izzy et al., 2019). Also, single cell RNA-seq data of FACS-isolated cell populations demonstrated that microglia differentially express clusters of genes possibly corresponded to various biological processes such as host defense response, synaptic plasticity, lipid remodeling and membrane polarization over the long-term course of TBI (Makinde et al., 2020). Notably, these longitudinal transcriptional changes of microglia may be associated with development of life-long neurologic impairment after TBI, which deserves further delineation in future studies (Makinde et al., 2020). Taken together, available evidence informs that microglia play a pivotal role in both the neuropathogenic and neurorestorative processes following TBI. The molecular and cellular mechanisms underlying the good, the bad, and the dysregulated microglia in TBI, however, is still under uncovering.

## Microglia and Neuroinflammation: A Culprit of Traumatic Brain Injury-Induced Neurodegeneration

**FIGURE 2:**
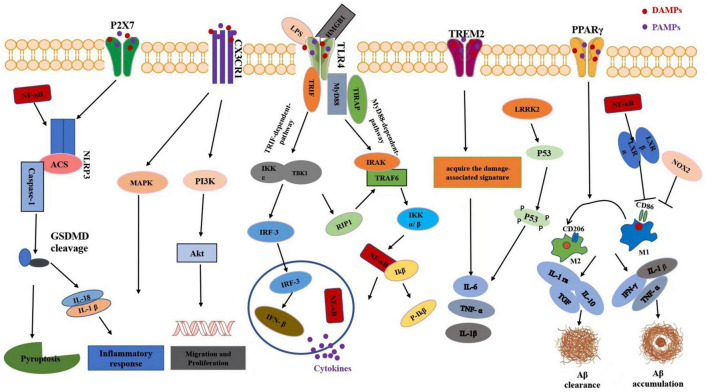
Schematic diagram of microglia-mediated neuroinflammation signaling pathways after TBI. In response to TBI, DAMPs such as HMGB1 can promote priming of the NLRP3 inflammasome through NF-κB signaling. In microglia, activation of P2X7 receptor could stimulate the NLRP3 inflammasome, which subsequently leads to the outflow of pro-inflammatory cytokines including IL-18 and IL-1β. CX3CL1 is also known to induce microglia activation through intracellular phosphorylation of microglial p38 MAPK. Further, treatment of cultured microglia with CX3CL1 promoted cell survival and inhibited Fas ligand-induced cell death via the Akt signaling pathway. TLR signaling initiates acute inflammatory response by promoting the release of pro-inflammatory cytokines. Ligand binding to TLR4 activates either a MyD88-dependent NF-κB activation or MyD88-independent activation of interferon regulatory factor-3 (IRF-3) activation and the subsequent expression of IFNβ. The TREM2 pathway is responsible for switching from a homeostatic to a neurodegenerative microglial phenotype after phagocytosis of apoptotic neurons. Upon activation, the PPARs regulate inflammation via trans-reexpression of multiple inflammatory signaling systems such as NFκB, activator protein-1 (AP-1), signal transducer and activator of transcription (STAT) to result in M1/M2 microglia polarization and facilitate it toward an anti-inflammatory state. In LPS induced neuroinflammation, LRRK2 could phosphorylate p53 and promote the production of pro-inflammatory cytokine such as tumor necrosis factor (TNF)-α. Activation of NOX2 aggravated inflammatory-mediated neurodegeneration after TBI via inhibiting switch from M1-like activation to M2- like activation of microglia. Pro-inflammatory cytokines expressed by microglia, including interferon-γ, interleukin-1β, and tumor necrosis factor-α, can specifically stimulate Aβ accumulation while anti-inflammatory cytokines such as IL-1ra, IL-10, and TGF preclude formation of Aβ. HMGB1, high mobility group box 1; N-FκB, nuclear factor-kappa B; MAPK, mitogen-activated protein kinase; PI3K, phosphoinositide 3-kinase; IFN-β, IRF-3-inducing interferon-b. TREM2, triggering receptor expressed on myeloid cells 2. LRRK2, leucine-rich repeat kinase 2; LPS, lipopolysaccharide; PPARγ, peroxisome proliferator-activated receptor gamma. IL-1ra, interleukin-1 receptor antagonist; Aβ, beta amyloid.

As mentioned above, TBI is an intrinsically heterogeneous, multifaceted disease which can encompass a myriad of sensorimotor, cognitive, and emotional deficits. It is now becoming increasingly clear that TBI is a chronic, evolving, and perhaps lifelong disorder, but rather than a static neurological insult. In particular, TBI has been identified as a significant risk factor for the development of AD, PD, CTE as well as other neurodegenerative diseases (Graham and Sharp, 2019). Pathological investigations revealed that diverse cellular and molecular mechanisms are involved in the pathophysiological processes of TBI, which favor significant contributions to chronic neurodegeneration after injury. These neuropathological cascades after TBI include neurotoxic proteinopathies related to accumulation of β-amyloid (Aβ), Tau, α-synuclein, and transactive response DNA binding protein (TDP-43), gliosis and neuroinflammation, BBB dysfunction and vascular pathology, as well as axonal degeneration and neuronal loss, among others. Specifically, as the most dominant immune cells in the brain, microglia constitute significant neuropathophysiological mechanisms in TBI, which also underlie a possible link between TBI and late-onset neurodegenerative diseases (Benarroch, 2013; Colonna and Butovsky, 2017). More importantly, proportions of crucial molecules regulate the activation and properties of microglia, which may serve as cellular players that shape evolving neurodegeneration following TBI (Puntambekar et al., 2018; Younger et al., 2019). For instance, microarray and pathway analyses of TBI-induced gene expression in the rat hippocampus and cortex at acute, subchronic and chronic intervals (24 h, 2 weeks, and 1, 2, 3, 6, and 12 months) after injury revealed that long-term, coordinated changes in expression of genes belonging to canonical pathways associated with the innate immune response (i.e., NF-κB signaling, NFAT signaling, complement system, acute phase response, Toll-like receptor (TLR) signaling, and neuroinflammatory signaling) (Boone et al., 2019). Thus, in the following sections, we will survey how do these key molecules regulate the activities and functional responses of microglia in TBI pathology and explore their potential implications to chronic neurodegeneration after injury (Figure 2).

## Toll-Like Receptors

Toll-like receptors are the best-characterized family of pattern-recognition receptors (PRRs) that are capable of initiating signals in response to diverse pathogen-associated molecular patterns (PAMPs) and tissue damage-associated molecular patterns (DAMPs) (Takeuchi and Akira, 2010). These evolutionarily conserved type I transmembrane proteins comprise an extracellular domain that recognizes exogenous and endogenous danger molecules and an ectodomain that activates downstream pathways in innate and adaptive immunity (Bruno et al., 2018). In the CNS, TLRs have been identified in different brain cell types, including microglia, astrocytes, oligodendrocytes and neurons. Specially, they may play a crucial role in the induction and regulation of microglial activation, not only in innate immunity in response to infectious conditions, but may also in CNS autoimmunity, tissue injury, and neurodegeneration. Upon stimulation, the TLR signaling can elicit the production of cytokines, enzymes and other inflammatory mediators in microglia, which can exert either beneficial or detrimental effects in the CNS depending on the context of tissue homeostasis or pathology.

During TBI, both the mRNA and protein levels of different TLRs, such as TLR2 and TLR4, are significantly increased in microglia in the cortex adjacent to the injured site in the acute stage after injury (Chen et al., 2009; Hua et al., 2011). In a weight-drop model of TBI, Zhu et al. (2014) reported that TLR4 is an important mediator in initiating the acute inflammatory response following TBI, and inhibition of the TLR4/MyD88/NF-κB signaling cascade by curcumin can reduce activation of microglia/macrophages, suppress inflammatory mediator release, and attenuate secondary brain injury at 24 h post-trauma. Chen et al. (2017) found that increased HMGB1 expression and extracellular release stimulates TLR4-mediated microglial activation and subsequent neuroinflammation in a rat model of TBI, while Omega-3 polyunsaturated fatty acid supplementation attenuates these neuroinflammatory responses and facilitates neurofunctional recovery at day 7 after TBI by inhibiting the HMGB1/TLR4/NF-κB pathway. Data from *TLR4* knockout (KO) mice also supported the crucial role of TLR4 signaling pathway in regulating microglial phenotype and activity following TBI (Yao et al., 2017). Specifically, TLR4 deficiency decreases MyD88/NF-κB but increases RAC-1/AKT activity, facilitates microglial M2 polarization and cell migration, and promotes inflammation resolution and neurological restoration after TBI (Yao et al., 2017). In a rodent model of repeated mTBI, TLR4 stimulation can exert either detrimental or beneficial effects depending on the timing of activation (Corrigan et al., 2017). Compared to lipopolysaccharide (LPS) administration at 1 day post-injury, injection of this TLR4 agonist at 5 days post-injury contributes to increased pro-inflammatory cytokine production and exacerbates neuronal damage following mTBI (Corrigan et al., 2017). Moreover, LPS administration at 5 days after repeated mTBI not only increases hyperphosphorylation and aggregation of Tau but also exacerbates neurofunctional deficits at 3 months post-injury, particularly in cognitive impairment and depressive-like behavior (Corrigan et al., 2017).

Indeed, the TLR signaling provides an important mechanism in regulating microglial responses over the course of chronic neurodegenerative diseases via modulating the production of inflammatory molecules and cellular phagocytosis (Lehnardt, 2010; Arroyo et al., 2011). On the one hand, appropriate activation of TLR receptors in microglia can exert protective effects and thus counteract the onset and progression of neurodegenerative pathologies via facilitating phagocytic clearance of protein aggregates and cellular debris (Venezia et al., 2017; Pourbadie et al., 2018). For instance, early minor stimulation of microglial TLR2 and TLR4 receptors by their ligands monophosphoryl lipid A (MPL), and Pam3Cys can facilitate microglia to sense/clear soluble Aβ, safeguard synaptic function, and improve AD-related memory deficits in rats (Pourbadie et al., 2018). Stimulation of the innate immune system and microglia/macrophage activation via TLR9 using CpG (cytosine phosphate-guanine) oligodeoxynucleotides (ODNs) also ameliorates vascular amyloid pathology and confers cognitive benefits in Tg-SwDI mice (Scholtzova et al., 2017). Chronic systemic treatment with monophosphoryl lipid A, a TLR4 selective agonist and a potent inducer of phagocytosis which does not trigger strong toxic inflammatory responses, leads to increased uptake of α-syn by microglial cells, rescue of nigral dopaminergic and striatal neurons, and amelioration of motor deficits in transgenic PLP-α-syn mice (Venezia et al., 2017). On the other side, aberrant activation of TLR signaling and release of pro-inflammatory cytokines in microglia are responsible for neurotoxic cascades and disease progression during chronic neurodegeneration (Jin et al., 2008; McDonald et al., 2016; Rubio-Araiz et al., 2018). Inhibiting TLR2 signaling can suppress glial activation, decreases Aβ plaque burden and improves behavior in spatial learning in a mouse model of AD (McDonald et al., 2016). By contrast, silencing of brain low-density lipoprotein receptor-related protein 1 (LRP1) markedly induces TLR4-mediated NF-κB and MAPK signaling pathway activation, increases microgliosis and astrogliosis, enhances pro-inflammatory cytokines production (i.e., TNF-α, IL-1β, and IL-6), exacerbates Aβ accumulation and synaptic and neuronal loss, and finally aggravates learning and memory deficits in APP/PS1 mice (He Y. et al., 2020). These findings highlight the complexity of the microglial and immune response in the pathophysiology of TBI and later developed neurodegeneration, which needs further investigation.

## NLRP3

The NOD-like receptor (NLR) family of proteins are a group of cytosolic PRRs known to mediate the initial innate immune response to cellular injury and stress. Among those NLRs, NLRP3 is one of the most well-studied members and mainly expressed in microglia (Xu et al., 2018). This sensor molecule can interact with the adapter protein ASC [apoptosis-associated speck-like protein containing caspase recruitment domain (CARD)] and the effector molecule pro-caspase-1 to form NLRP3 inflammasome. Once activated, the NLRP3 inflammasomes facilitate the cleavage and activation of caspase-1, which in turn mediates the cleavage of the pro-inflammatory cytokines IL-1β and IL-18 into their active and secreted forms. Besides, activated caspase-1 drives the cleavage of gasdermin D, which triggers an inflammatory form of cell death known as pyroptosis. Of note, the NLRP3 inflammasome activity is a necessary component of the innate immune response to tissue damage, and the functional regulation of an active NLRP3 inflammasome consists of two steps: a non-activating “priming” stimulus is firstly required to initiate expression of key inflammasome components, followed by a secondary “activating” stimulus that results in inflammasome oligomerization and activation.

Notably, dysregulation of NLRP3 inflammasome activity has been implicated in a number of neurological conditions, including acute brain injury and chronic neurodegeneration (Yi et al., 2019). In response to TBI, DAMPs such as high mobility group box 1 (HMGB1) can promote priming of the NLRP3 inflammasome through NF-κB signaling (Wang et al., 2018; Bao et al., 2019). In addition, TBI can produce a range of activating signals, including but not limited to the following: K^+^ efflux (Chen et al., 2019), mitochondrial ROS generation (Ma et al., 2017a), and cathepsin release after lysosomal destabilization (Zhou et al., 2018). As a consequence, the priming and activating stimuli trigger the activation of NLRP3 inflammasome following TBI, which play a key role in generating a significant neuroinflammatory response after injury (Ma et al., 2017a). Selective inhibition of NLRP3 inflammasome by small-molecule inhibitor such as MCC950 and JC124 can suppress microglial activation, reduce pro-inflammatory cytokines production and improve long-term motor and cognitive functions in murine models of TBI (Xu et al., 2018; Kuwar et al., 2019). Besides, NLRP3 has emerged as a prominent contributor to various neurodegenerative conditions, such as AD, PD, amyotrophic lateral sclerosis, and multiple sclerosis. Toxic Aβ peptide can light a fire in NLRP3 inflammasome and eventually induce AD pathology and tissue damage, while inhibition of NLRP3 could largely decrease Aβ deposition, alleviate deterioration of neuronal function, and protect from memory loss, cognitive function in AD transgenic mouse model (Tan et al., 2013; Dempsey et al., 2017). Also, activation of microglial NLRP3 inflammasome, a common pathway triggered by both fibrillar α-synuclein and dopaminergic degeneration, is a sustained source of neuroinflammation that could drive α-synuclein pathology and progressive dopaminergic neurodegeneration (Gordon et al., 2018). In the 1-methyl-4-phenyl-1,2,3,6-tetrahydropyridine (MPTP) mouse model of PD, neurotoxin MPTP activates NLRP3 inflammasome signaling to promote microglial recruitment, caspase-1 activation and interleukin-1β production in the substantia nigra of suffered brain, which is critical for dopaminergic neuronal loss and the subsequent motor deficits (Lee E. et al., 2019). Therefore, these findings suggest that microglial NLRP3 inflammasome signaling might serve as a therapeutic target for preventing the progression of chronic neurodegeneration after TBI.

## Chemokine Receptors CX3CR1

The chemokines comprise a family of small secreted proteins that regulate cell migration and chemotaxis, survival and proliferation via activation of chemokine receptors. They can bind and initiate that signal through cell surface G-protein coupled heptahelical chemokine receptors. Among more than 50 known chemokines, fractalkine is the sole member of the CX3C family, and has unique structural and functional attributes (Hughes and Nibbs, 2018). Fractalkine, also known as CX3CL1, is the only member of the ∂ (CX3C) chemokine family, characterized by the presence of 3 amino acidic residues (X3) localized between two cysteine residues, thus forming a disulfide bond, a CX3C motif, and also a transmembrane domain. They are best known for their ability to stimulate the migration of cells, most notably white blood cells (leukocytes). Consequently, chemokines play a central role in the development and homeostasis of the immune system, and are involved in all protective or destructive immune and inflammatory responses. Chemokine receptors belong to the family of G-protein coupled receptors (GPCR), showing the presence of 7 transmembrane helices connected by several intra- and extracellular loops, as well as N-terminal extracellular and C-terminal intracellular domains. The fractalkine receptor CX3CR1 is a Gi-protein coupled receptor encoded by the *Cx3cr1* gene. Unlike most ligands belonging to the chemokine family with the affinity for numerous receptors, CX3CL1 reveals its biological activity via an interaction with only one dedicated receptor—CX3CR1 (Poniatowski Ł. et al., 2017). Both membrane-bound and soluble CX3CL1 bind a unique G protein–coupled receptor, CX3CR1, that in the brain is restricted to microglial cells (Harrison et al., 1998). Fractalkine (CX3CL1) is an intriguing chemokine that plays a central role in the nervous system. The expression of CX3CL1 on neurons and expression of its receptor CX3CR1 on microglia facilitates a privileged interaction, playing important roles in regulating the function and maturation of these cells. CX3CL1 is reported to have neuroprotective and anti-inflammatory activities in several experimental systems and animal models of diseases, and its expression correlates with positive outcomes in human neuropathologies. However, a comparable amount of evidence shows that CX3CL1 sustains neuroinflammatory conditions and contributes to neurotoxicity. The fractalkine (CX3CL1)/CX3CR1 pair represents an interesting path of signaling between neurons, microglia, and astrocytes in different aspects of CNS function (Lauro et al., 2015).

## Triggering Receptor Expressed on Myeloid Cells 2

Triggering receptor expressed on myeloid cells 2 (TREM2) is a transmembrane receptor mainly expressed in myeloid lineage cells, including peripheral macrophages, granulocytes, dendritic cells, osteoclasts, and microglia in the CNS (Kober and Brett, 2017). This cell-surface receptor belongs to the superfamily of immunoglobulin with an important role in the regulation of bone homeostasis, neurological development, and inflammation (Wolfe et al., 2018; Deczkowska et al., 2020). Studies showed that expression of TREM2 is upregulated in a wide range of diseases, namely AD, PD, TBI, stroke, and amyotrophic lateral sclerosis (ALS) (Gratuze et al., 2018). In the CNS, activation of TREM2 was reported to transform the microglia from homeostatic state to disordered state (Forabosco et al., 2013), accompanied by functional changes to microglia such as proliferation, migration, cytokine production, cellular metabolism, and phagocytosis (Ulland et al., 2017; Gratuze et al., 2018; Zheng et al., 2018). TREM2-mediated signaling pathway in microglia has also functioned in the process of inflammation, in which inhibition of TREM2 leads to increase of TNF-α and NO synthase-2 transcription (NOS2), while activation of TREM2 results in reduction of NOS2 and TNF-α expression (Takahashi et al., 2005). TREM2 performs as a neuro-protective character in cerebral disorders or injury. Gene expression data identified *Trem2* as a key hub in mice brain after TBI, with upregulated expression of TREM2 protein (Castranio et al., 2017). In a mouse model of TBI employing lateral fluid percussion injury (FPI), researchers found that deficiency of TREM2 affects functional and pathological outcomes of mice at early and late stages after TBI (Saber et al., 2017). In neurodegenerative diseases such as AD, multiple TREM2 variants in microglia have also been reported from human data to play a role, loss-of-function of which is closely link to elevated risk of developing the disorders (Guerreiro et al., 2013; Ulland and Colonna, 2018). Furthermore, recent study revealed that genetic knockout of *TREM2* in mice regulates deposition of Aβ species in extracellular plaques, as well as intraneuronally, in line with the findings from AD patients (Joshi et al., 2021).

## Peroxisome Proliferator-Activated Receptor Gamma

Peroxisome proliferator-activated receptor gamma (PPARγ) is a type of cellular transcriptional factor activated by ligands belonging to the superfamily of nuclear hormone receptor (NHR) (Prashantha Kumar et al., 2020). PPARγ is mainly regarded as a critical modulator of important genes in the processes of metabolism and cell differentiation. In addition, PPARγ is also capable of repressing other transcription factors, including NF-κB, Stat 1, and transcription factor activator protein-1. Besides, PPARγ has been implicated in the downregulation of iNOS, MM9, and COX2, indicative of a promising role in inflammation (Lenglet et al., 2015). In the CNS, PPARγ is widely distributed in various cell types, such as microglia, astrocytes, neurons, and oligodendrocytes. A group of studies reported that activation of PPARγ results in M1/M2 microglia polarization and facilitates it toward an anti-inflammatory state (Han et al., 2015; Hu et al., 2015; Pan et al., 2015). PPARγ-dependent anti-inflammatory functions participate in the process of TBI or neurodegenerative disorders (Villapol, 2018). In the mouse models of TBI, upregulation of PPARγ promotes the transformation of microglia from M1 to M2 polarization, ameliorates brain injury, as well as neurological functions. Nevertheless, application of PPARγ antagonist or inhibitor could reverse the neuroprotective effects (He et al., 2018; He J. et al., 2020; Jiang et al., 2020, 2021). In a wide array of neurodegenerative diseases, including AD, PD, ALS, activation of PPARγ has been found beneficially regulated by various ligands or natural substances (Mirza et al., 2019). For instance, in the onset and disease progression of PD, expression of PPARγ is attenuated along with activated cell death and upregulated oxidative stress, which can be reversed by PPARγ agonists, indicating the neuroprotective role of this transcription factor (Schintu et al., 2009; Falcone et al., 2015). Furthermore, several recent studies showed that PPARγ agonist could also improve neurodegeneration induced by TBI via promoting neuron survival, which lead to novel hypothesis that PPARγ might exert critical functions in the pathogenesis of neurodegeneration following TBI (Pilipović et al., 2015; Liu M. et al., 2017).

## Liver X Receptor

Liver X receptor (LXR) is a ligand activated transcription factor belonging to the family of nuclear receptors. There are two subtypes of LXRs including LXRα and LXRβ, the former subtype is predominantly distributed in adipose tissue, liver, and brain, while the latter subtype is widely expressed in the whole body, particularly in the brain (Bu, 2009). In the CNS, LXR exists widely in glia cells and mature or developing neurons (Mandrekar-Colucci and Landreth, 2011). LXR mainly exerts its functions in the process of lipid metabolism, as well as cholesterol homeostasis (Bogie et al., 2021). Activation of LXR participates in cholesterol metabolism via modulating relevant genes expression. Furthermore, recent studies also revealed that LXR plays a critical role in inflammatory responses with influence on immune cells. Through transrepressing activator protein-1 and NF-κB, the inflammatory phenotype of microglia and macrophages could be downregulated upon LXR activation (Ghisletti et al., 2007; Mouzat et al., 2019). In microglia, several *in vitro* and *in vivo* studies have demonstrated that activation of LXR reduces production of reactive nitrogen species, and microglia activation could be inhibited by LXR (McVoy et al., 2015; Wu et al., 2016; Paterniti et al., 2017). As a result, appropriate LXR ligands selectively targeting microglia-dependent pathological process might be a potential strategy to regulate neuroinflammation, especially in neurodegenerative disorders (Fessler, 2018). *Lxr* deficient mice presented motor phenotype similar to symptoms of ALS patients (Andersson et al., 2005). Recent human gene data confirmed that LXR isoforms, LXRα and LXRβ, function as genetic modulators in ALS patients (Mouzat et al., 2018). In a transgenic mice model of AD, scientists found that activation of LXR in microglia disturbs the NF-κB signaling pathway via decreasing generation of related pro-inflammatory factors (Cui et al., 2012). In the study of multiple sclerosis (MS), LXRs have been implicated in the regulation of myelination and remyelination, through regulating the expression of genes that are imperative in formation of myelin sheath (Shackleford et al., 2017). Except neurodegenerative diseases, LXR-mediated anti-inflammatory response also plays a role in the condition of TBI. An animal model of TBI showed that expression of inflammatory cytokines is dramatically suppressed after use of LXR agonists (Yu et al., 2016). Furthermore, LXR activation by synthetic agonists have been reported to reduce Aβ accumulation, decrease lesion volume, lessen axonal impairment, and promote functional recovery in mouse models of TBI (Loane et al., 2011; Namjoshi et al., 2013).

## Leucine-Rich Repeat Kinase 2

Leucine-rich repeat kinase 2 (LRRK2) is one member of the ROCO protein family, which is named for Ras-of-Complex (Roc) domain and C-terminal-of-Roc (COR) domain. It is a large protein comprised of a Roc GTPase domain, a serine/threonine kinase domain, and several protein-protein interacting domains. Therefore, LRRK2 functions enzymatic activity as a GTPase or a kinase, and may also provide scaffold for protein assembly (Islam and Moore, 2017). LRRK2 is widely expressed in different organs, particularly in the brain including neurons and microglia. In microglia, *in vitro* and *in vivo* studies indicated that LRRK2 protein is expressed robustly after lipopolysaccharide (LPS) induction (Gillardon et al., 2012; Moehle et al., 2012). It was reported that LRRK2 participates in the process of neuroinflammation through microglia activation (Lee et al., 2017). In LPS induced neuroinflammation, LRRK2 could phosphorylate p53 and promote the production of pro-inflammatory cytokine such as tumor necrosis factor (TNF)-α (Ho et al., 2017). *Lrrk2* knockdown or pharmacological kinase inhibition in primary microglia was reported to attenuate pro-inflammatory signals, including secretion of TNF-α, iNOS induction, and NF-κB transcriptional activity (Gillardon et al., 2012; Kim et al., 2012; Moehle et al., 2012). In neurons, endogenous LRRK2 could be induced by TBI through the mechanism of hypoxia induced factor (HIF) 1α-dependent transcriptional activation. Various types of TBI models indicated that LRRK2 worsens neuronal cell death, neuroinflammation, and behavioral deficits induced by brain injury, which could be alleviated after application of LRRK2 inhibitor (Bae et al., 2018). Furthermore, LRRK2 could also induce secondary brain injury after TBI through p38/Drosha signaling pathway in a rat model (Rui et al., 2018). In addition, LRRK2 expression is also found in immune cells and is closely linked to neuroinflammation in PD (Alessi and Sammler, 2018). Mutations in *Lrrk2* gene are highly connected with autosomal dominant familial PD (Singleton and Gasser, 2020). Many models of PD indicated that knockdown or pharmacological inhibition of *Lrrk2* gene could be beneficial to the disease (Chen et al., 2018). A subset of Rab GTPases closely connected with late onset PD were identified as substrates of LRRK2 (Steger et al., 2016), suggesting that in PD induced by TBI, TBI may elevate the expression of Rab proteins in brain and then increase disrupted LRRK2 substrates in glial cells and neurons which finally lead to PD.

## P2X7 Receptor

P2X purinoceptor family are a group of ligand-gated ion channel receptors which open or close dependent on extracellular level of adenosine triphosphate (ATP) (Sperlágh et al., 2006). There are seven subtypes of P2X purinoceptors, namely P2X1, 2, 3, 4, 5, 6, and 7, among which P2X7 receptor is generally activated by moderately high level (>100 μM) of ATP generated by pathological processes, whereas other P2X receptor subtypes are stimulated by lower level of ATP (Di Virgilio, 2021). P2X7 receptor is widely distributed in different cell types of peripheral tissues and central nervous system (Burnstock, 2008). In the CNS, P2X7 receptor is predominantly expressed in glia cells including microglia, oligodendrocytes, and astrocytes (Bhattacharya and Biber, 2016). In microglia, activation of P2X7 receptor could stimulate the NLRP3 inflammasome, which subsequently leads to the outflow of pro-inflammatory cytokines including IL-18 and IL-1β, the pivotal neuroinflammation mediators (He et al., 2017). P2X7 receptor is also involved in the pathological condition of TBI, in which P2X7 receptor in microglia of the cerebral cortex is reported up-regulated. After application of P2X7 receptor immune inhibitor (FTY720) and antagonist (A804598) in a TBI model, increased number of neurons survive with apoptotic cell death reduced in the injured cerebral regions (Liu X. et al., 2017). Another study also indicated that P2X7 receptor attends the pathological process of cerebral edema and neurological damage after TBI. The level of aquaporin-4, a water channel of astrocyte and facilitating cellular edema, was detected reduced after P2X7 receptor was antagonized (Leeson et al., 2019). On the other hand, P2X7 receptor-mediated activation of microglia has been implicated to contribute to neurodegeneration through neuroinflammation (Illes et al., 2017). ATP-dependent activation of P2X7 receptor induced microglial proliferation and activation, which resulted in neuronal cell death, cellular excitotoxicity mediated by glutamate, and activation of NLRP3 inflammasome. Consequently, pro-inflammatory cytokines were generated and released, as well as reactive oxygen and nitrogen species, both leading to the condition of neurodegeneration (Territo and Zarrinmayeh, 2021). In an AD model study, it was showed that inhibition of P2X7 receptor with antagonists could reduce the level of amyloid plaque and hinder the progression of the disease, indicating P2X7 receptor as a potential therapeutic target for neurodegenerative diseases (Rodrigues et al., 2015).

## NADPH Oxidase 2

NADPH oxidase 2 (NOX2) is a multi-subunit enzyme system that catalyzes the generation of intracellular and extracellular reactive oxygen species (ROS) in phagocytes, mainly microglia and peripheral macrophages. The production of excessive ROS participates in various cellular processes including neuroinflammation, neurodegeneration and brain trauma (Simpson and Oliver, 2020). Activation of NOX2 has been reported to facilitate oxidative damage in CNS under pathological conditions of trauma, ischemia, and degeneration (Ano et al., 2010; Cairns et al., 2012). In addition, studies have demonstrated that pharmacological inhibition of NOX2 could restrain oxidative stress and inflammatory response, suggesting a promising therapeutic candidate in inflammatory disorders (Glennon-Alty et al., 2018). Single-cell RNA-seq data from human and murine brains has revealed that NOX2 (*CYBB*) is the most common expressed transcript of NOX isotypes in microglia (Zhang et al., 2014, 2016). Furthermore, NOX2 is expressed throughout the development of CNS in both human and mouse microglia, suggestive of a possible role in brain evolution (Simpson and Oliver, 2020). Microglial activation of NOX2 can lead to neurotoxicity, which mediated by the generation of extracellular ROS, and redox signaling pathway that promotes release of pro-inflammatory factors. Oppositely, disturbance of NOX2 expression in microglia brings about resistance to neurotoxicity, which is evident in midbrain cultures of *NOX2* knockout rats treated with α-synuclein and lipopolysaccharide (LPS) (Qin et al., 2013; Zhang et al., 2018). The neuronal toxicity mediated by NOX2 in microglia has also been implicated in the pathogenesis of TBI. In a mouse model of controlled cortical injury (CCI), mRNA expression of NOX2 was significantly increased in the region of injured cortex (Kumar et al., 2019). Immunofluorescence results have confirmed the robust expression of NOX2 in mechanical damage of both brain and spinal, which persisted beyond 28-day after injury (Bermudez et al., 2016). A variety of mouse models of TBI, employing genetic deletion tools or pharmacological treatments to suppress NOX2 expression, showed weakening of pro-inflammatory microglial responses with reduced production of cytokines, strengthened anti-inflammatory responses regulated by NOX2-mediated Redox signaling, and inhibition of oxidative stress and neurotoxicity (Kumar et al., 2016b; Barrett et al., 2017; Wang and Luo, 2020). Functionally, repression of NOX2 after TBI improved long-term cognitive and motor impairment in mice (Kumar et al., 2016a,b; Barrett et al., 2017; Ma et al., 2017b). Enhanced NOX2 activation is also implicated in a variety of neurodegenerative diseases. Experimental studies revealed NOX2-mediated neurotoxicity in microglia as an inevitable process throughout chronic neurodegeneration (Block et al., 2007). Pharmacological repression of microglial NOX2 attenuated LPS-induced neurodegeneration in a mouse model, even after the neurodegeneration had already been initiated (Wang et al., 2020). Similar results were also reported in research with *NOX-2* genetically knockout mice (Shahraz et al., 2021). In addition, inhibiting the activation of NOX2 with ginsenoside Rg1 could improve cognitive impairments, protect neurons from damage, and lower the deposition of amyloid beta in mice modeling Alzheimer’s disease (AD) (Zhang et al., 2021). Furthermore, NOX2-driven microglial activation has also been implicated in the process of TBI-induced neurodegeneration. With application of *NOX2*-knockout mice or medical inhibitor, researchers found that *NOX2* deficiency improved inflammatory-mediated neurodegeneration after TBI, via promoting switch from M1-like activation to M2- like activation of microglia (Kumar et al., 2016b).

## Conclusion and Future Perspectives

Neuroinflammation is proposed as an important manipulable aspect of secondary injury in animal and human studies. Because neuroinflammation can be detrimental or beneficial, before developing immunomodulatory therapies, it is necessary to better understand the timing and complexity of the immune responses that follow TBI. Importantly, the mechanisms underlying the pathogenesis that lead to such disabilities are still incompletely understood. Therefore, while the post-TBI central nervous system (CNS) illnesses have a high prevalence; few, if any, treatments are available to deter and prevent the pathological progression thought to lead to chronic neurological diseases and conditions. Thus, a better understanding of the molecular mechanisms underlying TBI and neurological diseases is crucial to uncover the potential link between these conditions to enable development of effective diagnostic and treatment strategies which could reduce the incidence of post-TBI neurological complications.

A comprehensive understanding of the relationship between TBI and neurodegenerative diseases will require development of novel animal models that enable us to study the factors that initiate β-amyloid (Aβ) accumulation as well as tau phosphorylation and aggregation. In addition, more advanced clinical neuroimaging [e.g., positron emission tomography (PET) imaging to detect pathological tau and amyloid deposition] is needed to explore the mechanisms driving chronic neurodegeneration after TBI in humans and selecting patients for potential future therapies.

The specific roles of polarized microglial and macrophage populations in CNS repair after acute injury, and argues that therapeutic approaches targeting cerebral inflammation should shift from broad suppression of microglia and macrophages toward subtle adjustment of the balance between their phenotypes. Breakthroughs in the identification of regulatory molecules that control these phenotypic shifts could ultimately accelerate research toward curing brain disorders.

## Author Contributions

FS, XW, and HW conceived the idea of the study and drafted the manuscript. QW and JZ polished the language and revised the manuscript. All authors read and approved the final version of the manuscript.

## Conflict of Interest

The authors declare that the research was conducted in the absence of any commercial or financial relationships that could be construed as a potential conflict of interest.

## Publisher’s Note

All claims expressed in this article are solely those of the authors and do not necessarily represent those of their affiliated organizations, or those of the publisher, the editors and the reviewers. Any product that may be evaluated in this article, or claim that may be made by its manufacturer, is not guaranteed or endorsed by the publisher.
